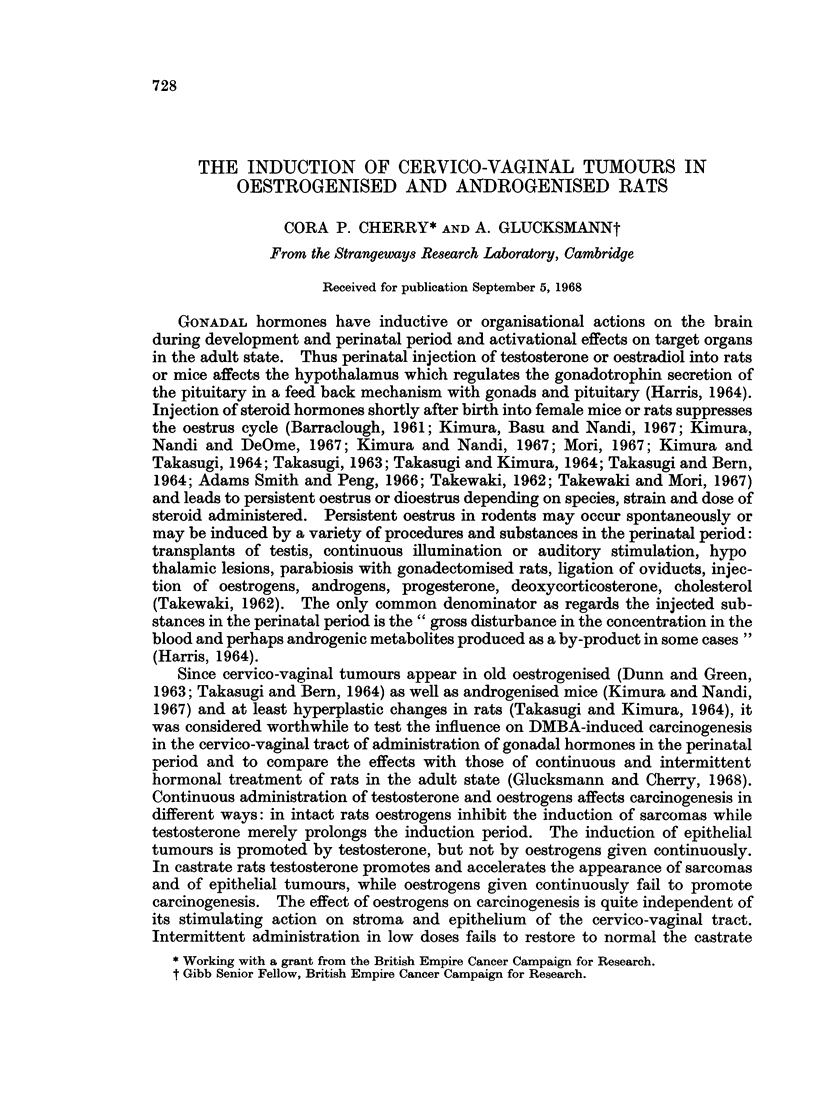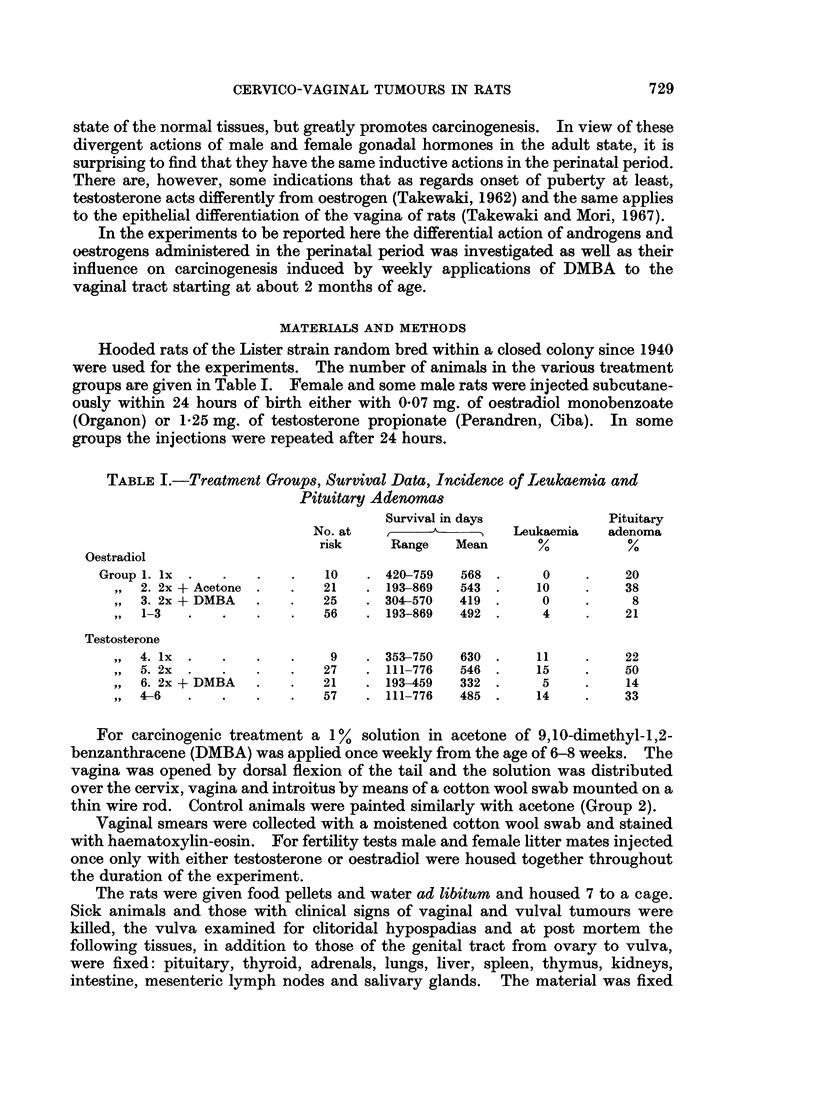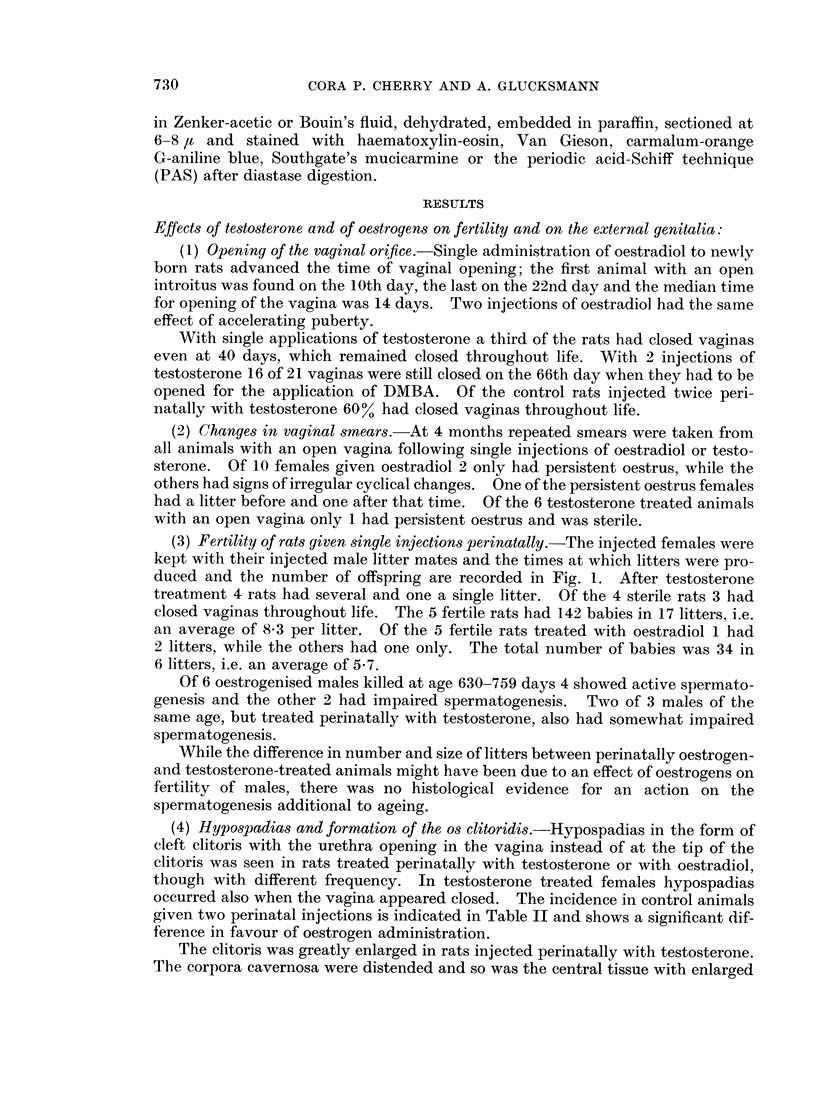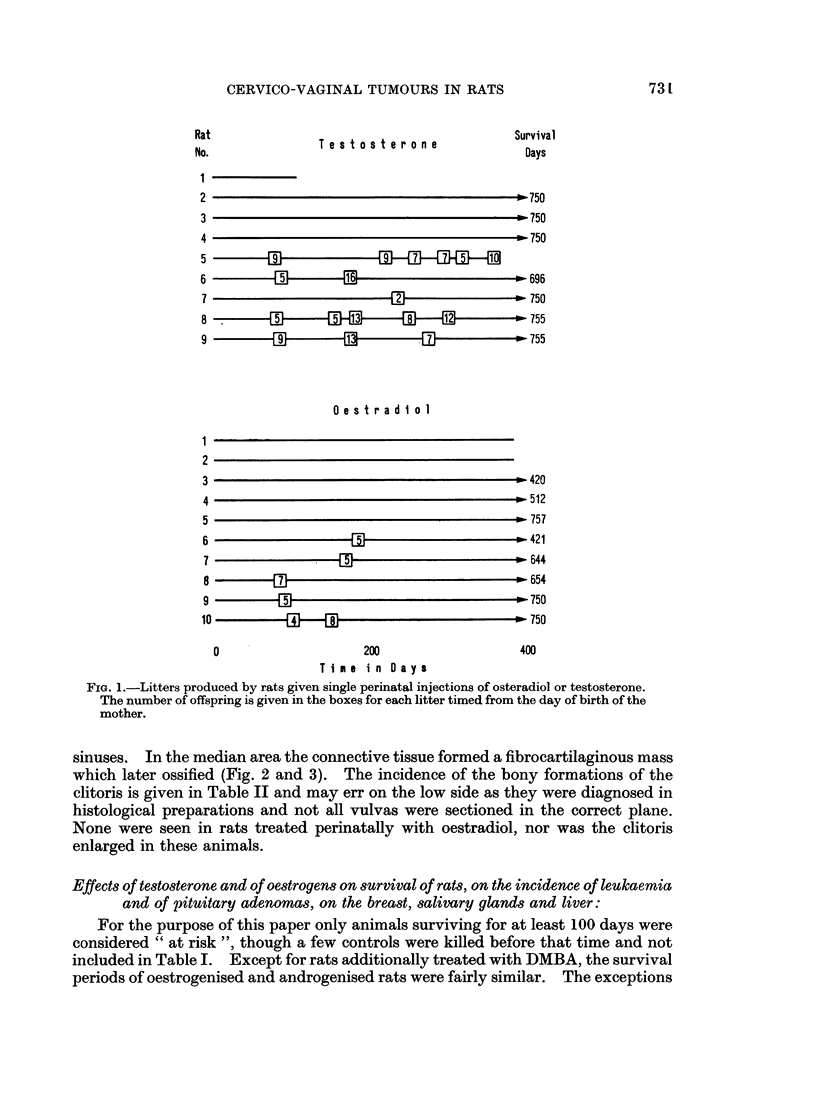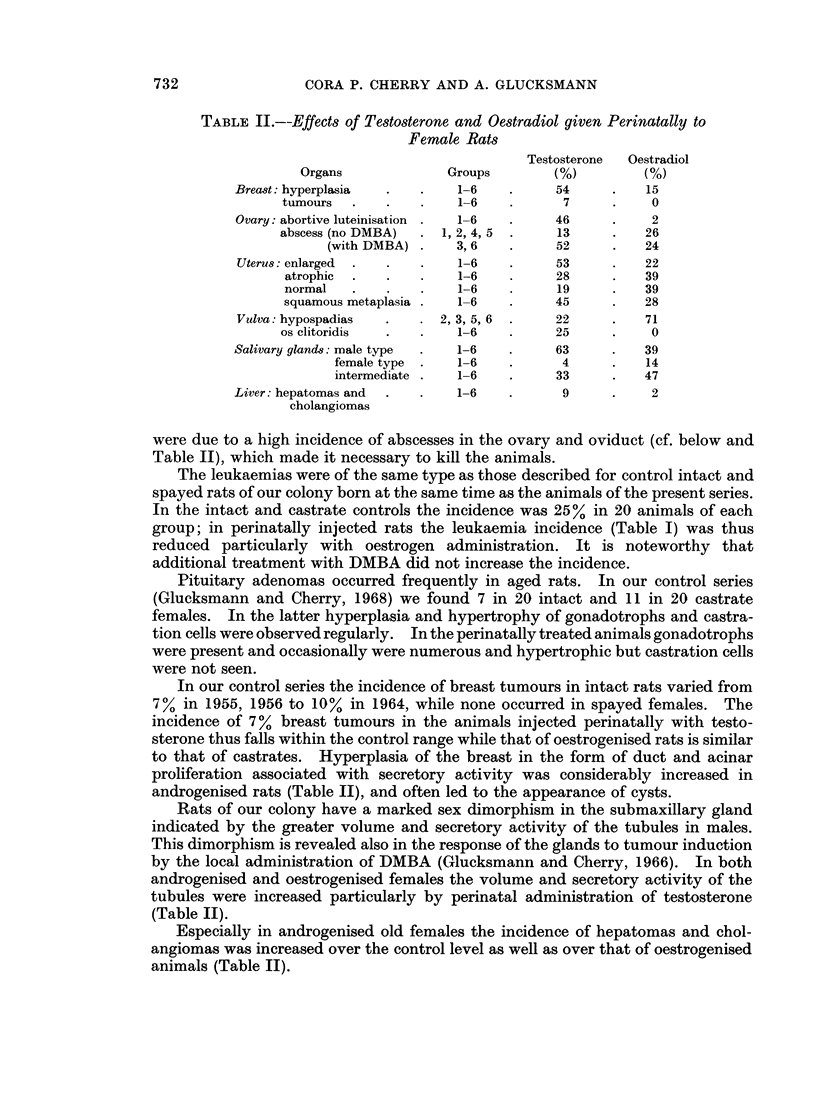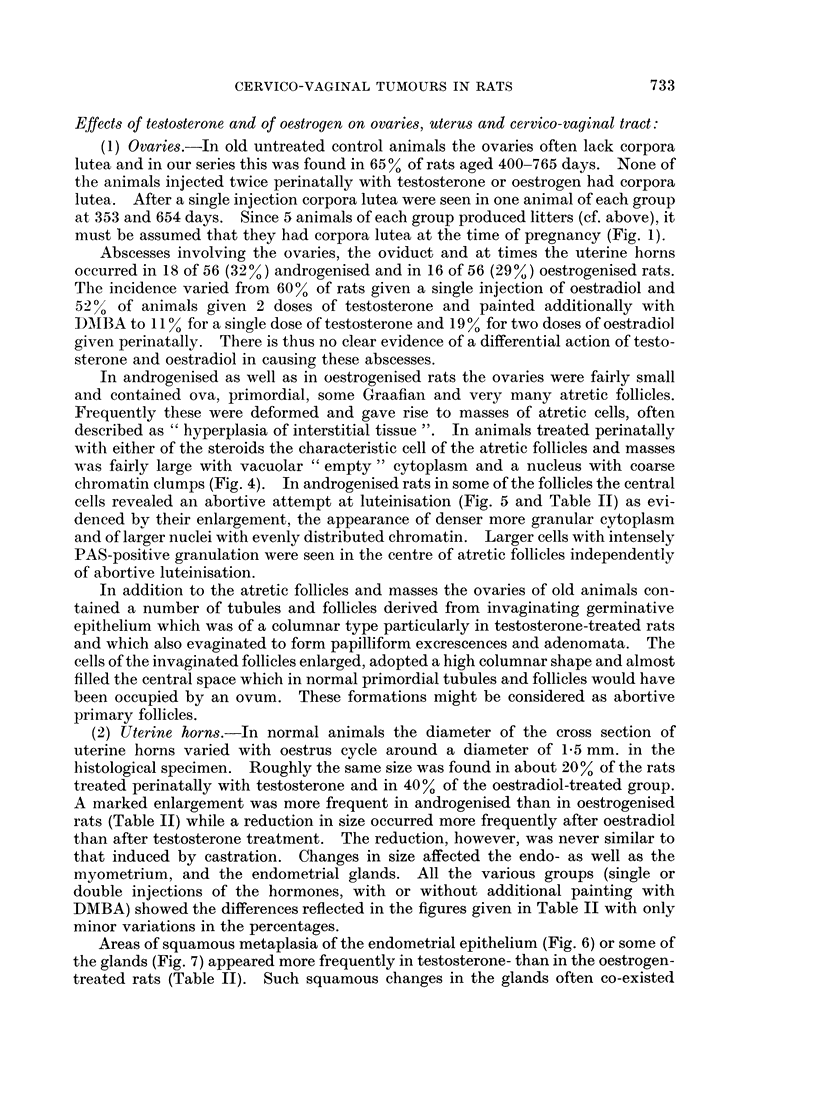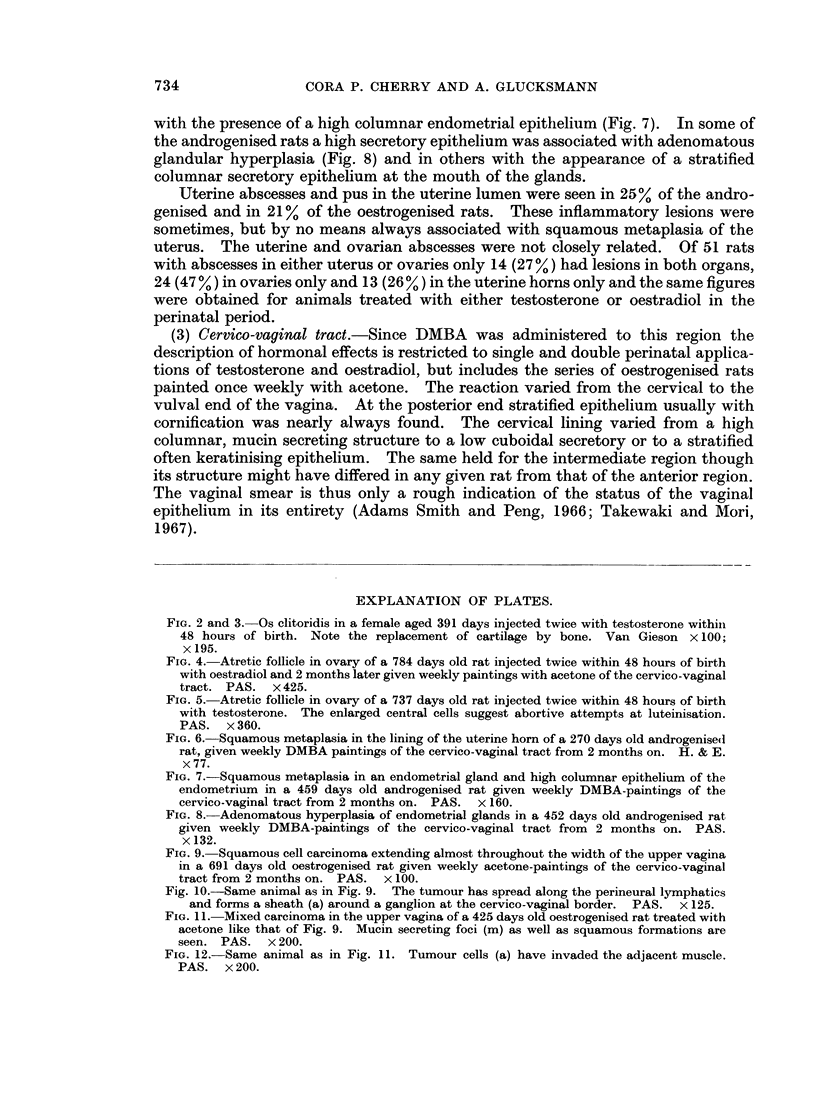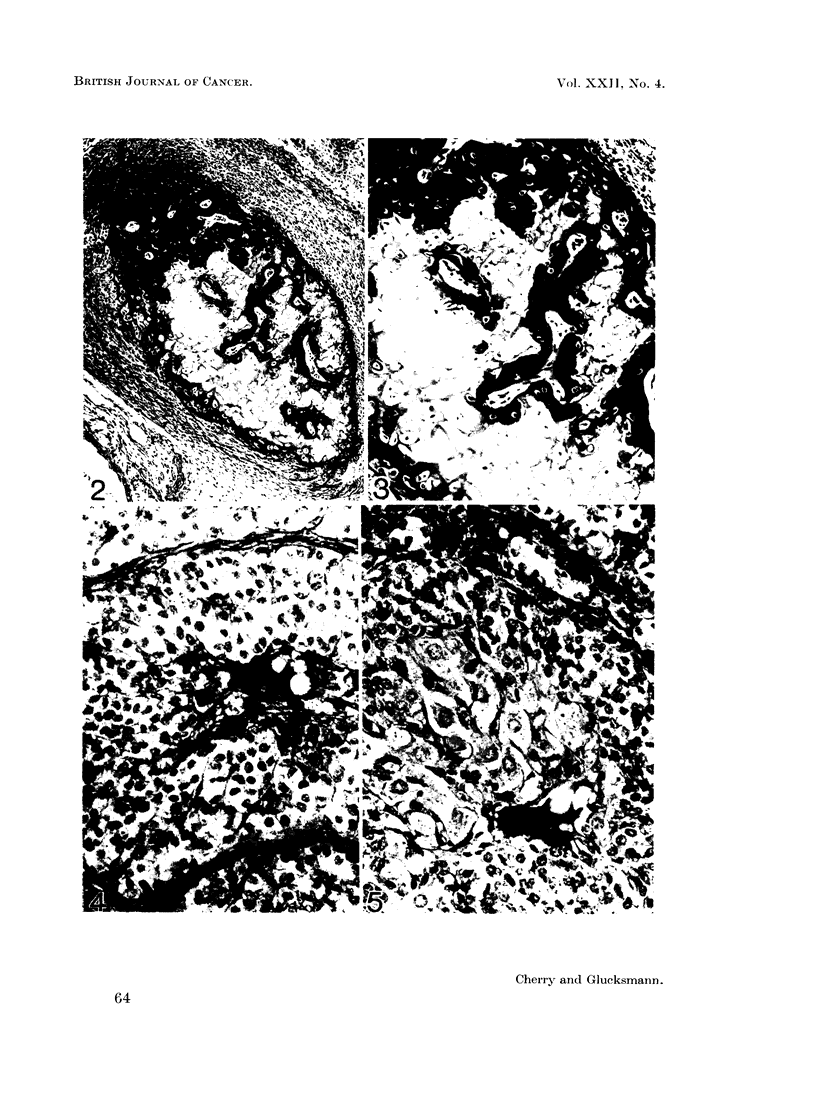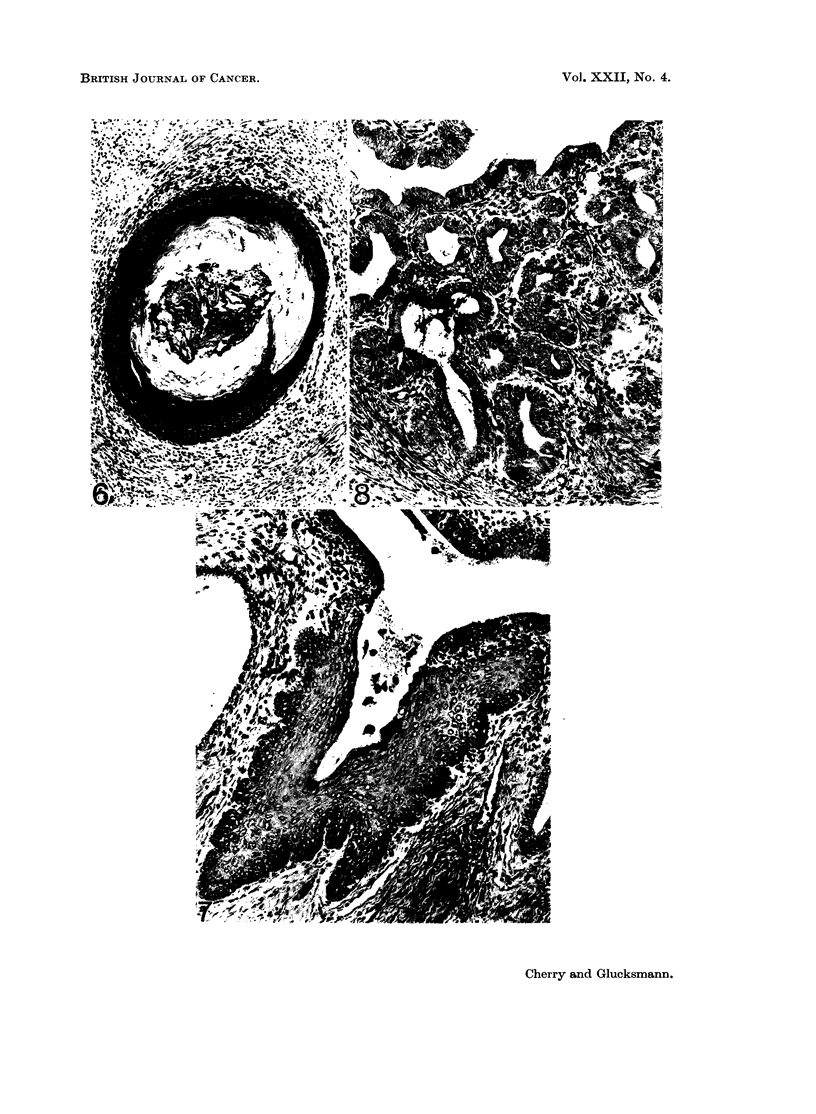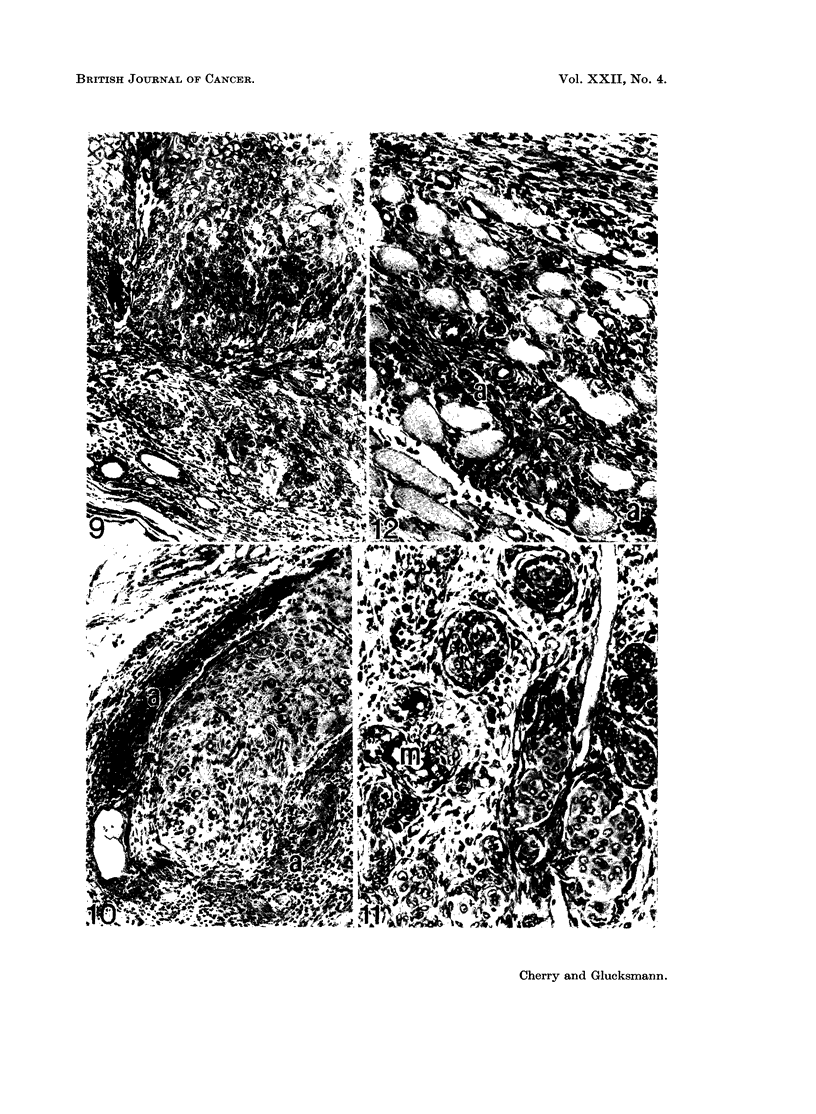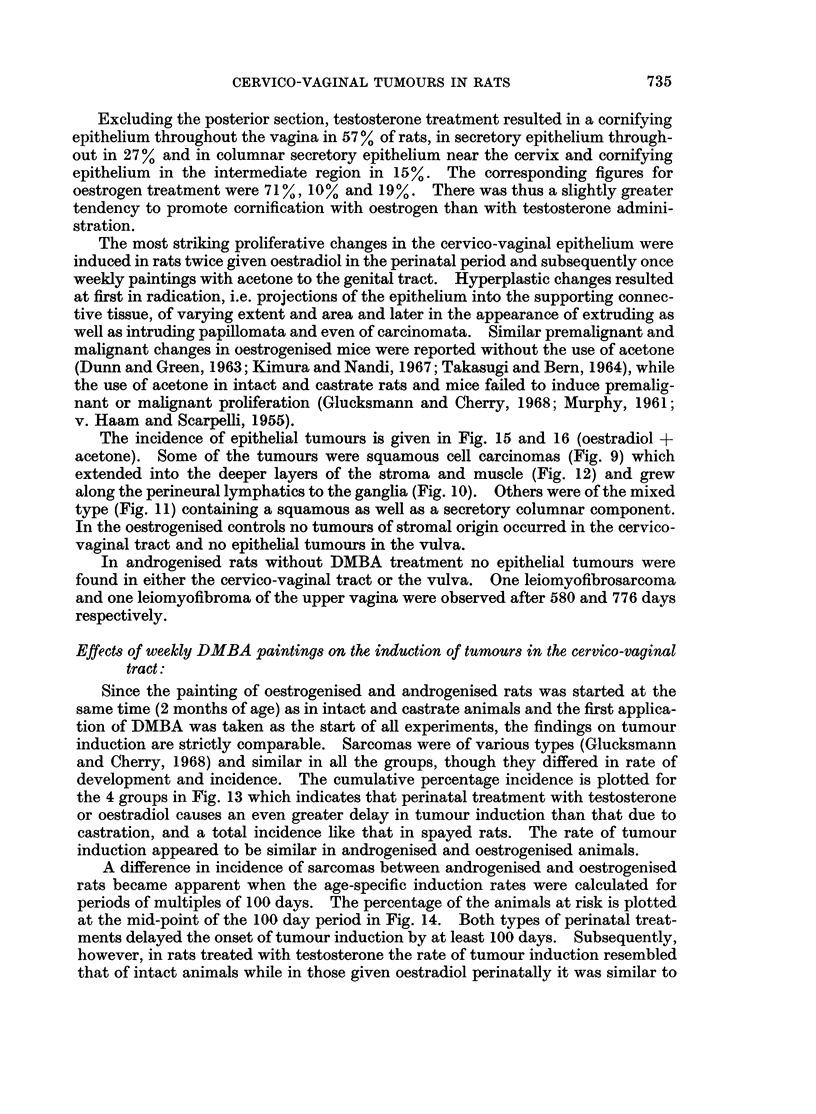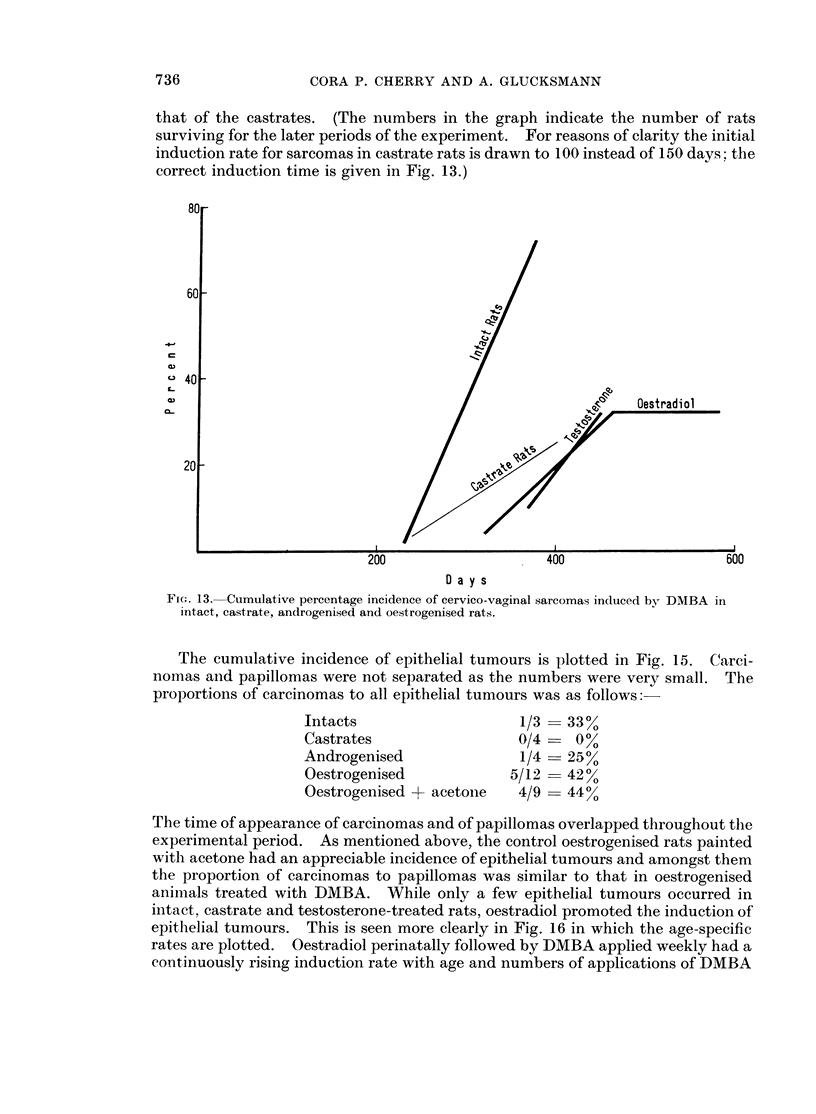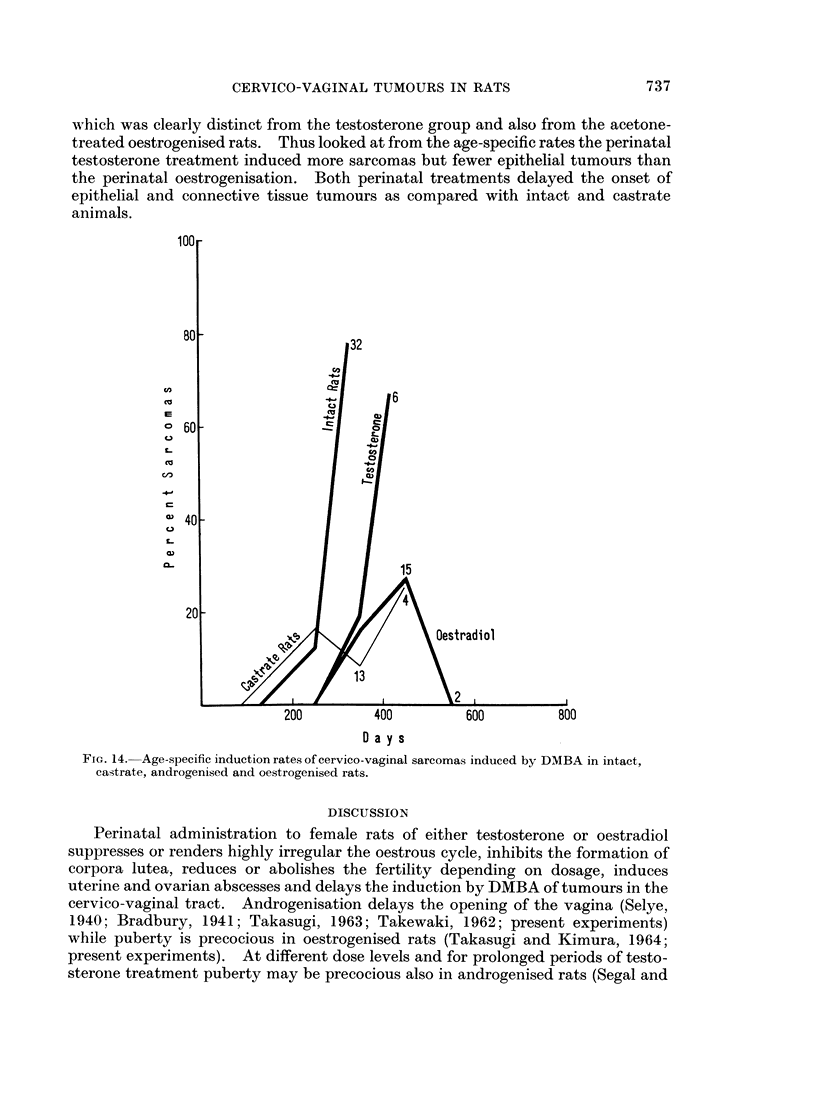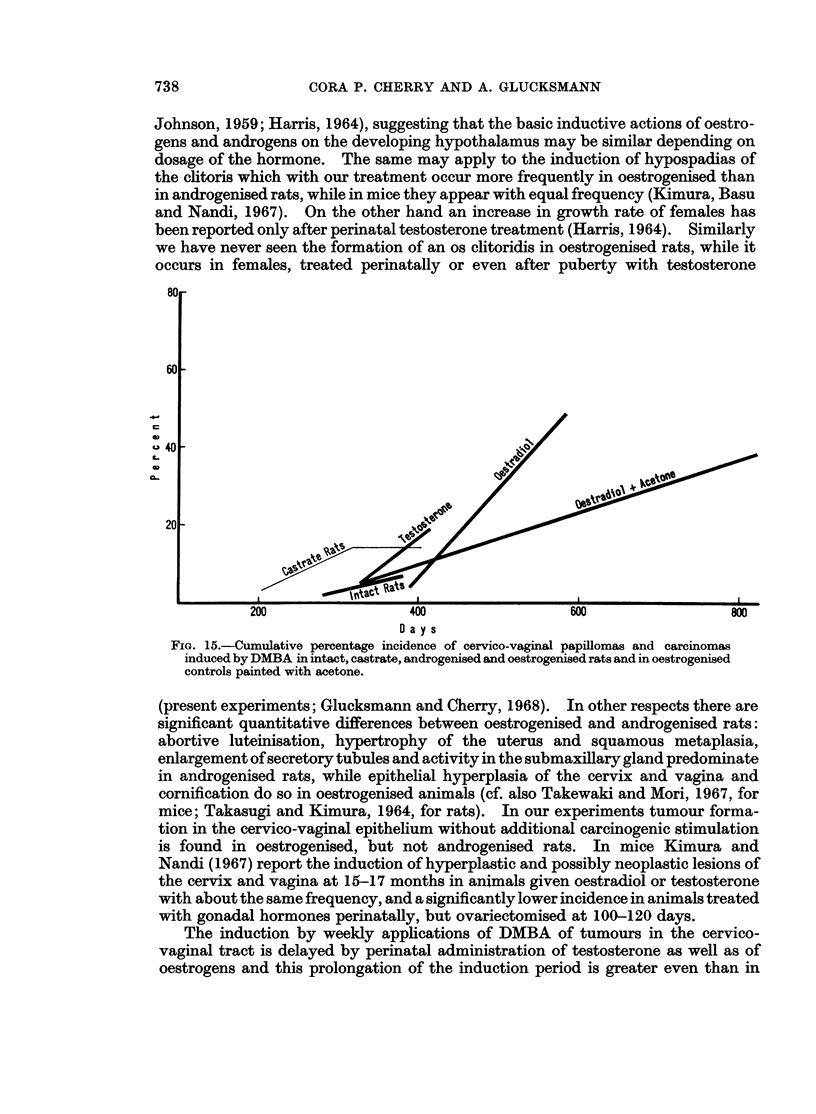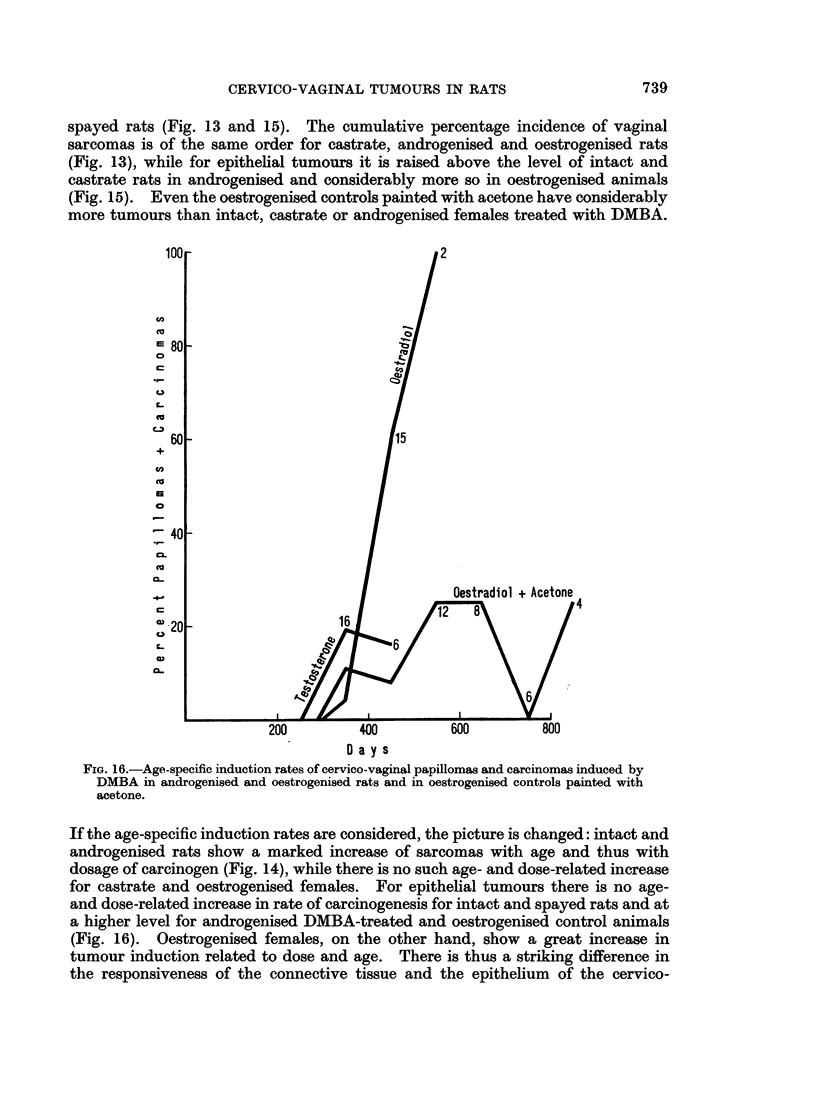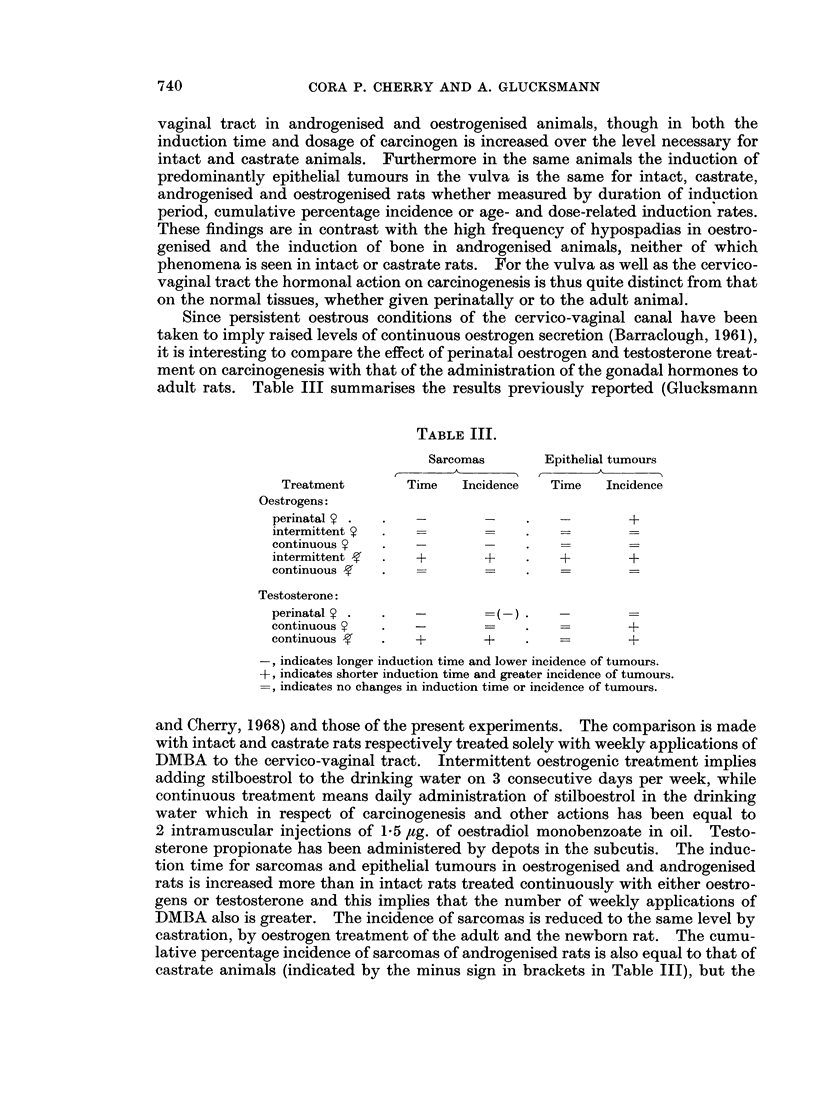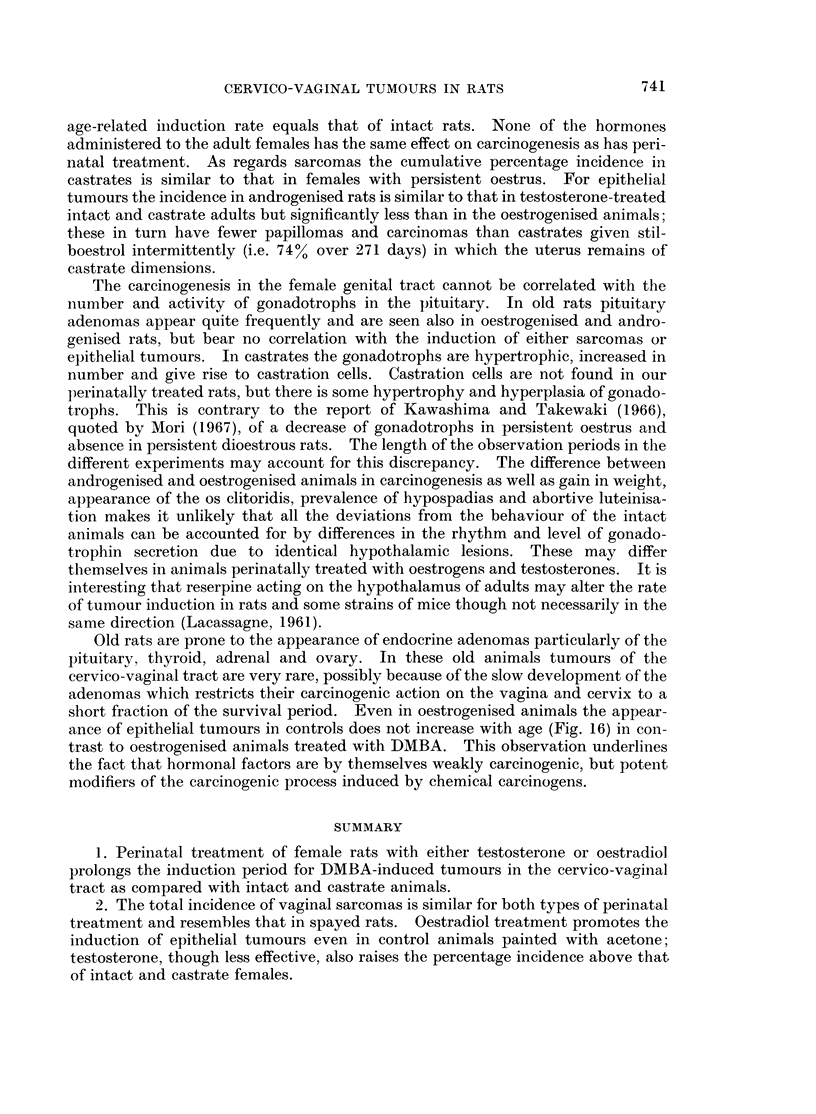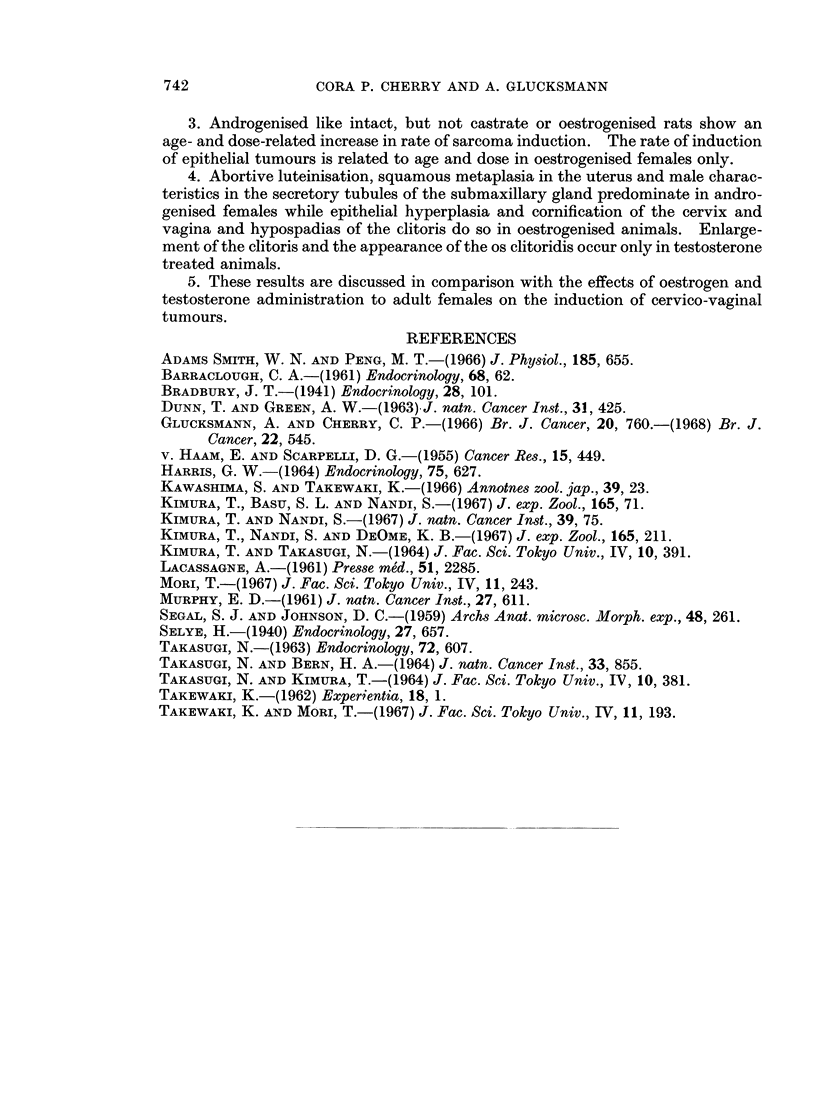# The induction of cervico-vaginal tumours in oestrogenised and androgenised rats.

**DOI:** 10.1038/bjc.1968.87

**Published:** 1968-12

**Authors:** C. P. Cherry, A. Glucksmann

## Abstract

**Images:**


### THE INDUCTION OF CERVICO-VAGINAL TUMOURS IN OESTROGENISED AND ANDROGENISED RATS

CORA P. CHERRY* AND A. GLUCKSMANN†

*From the Strangeways Research Laboratory, Cambridge*

Received for publication September 5, 1968

GONADAL hormones have inductive or organisational actions on the brain during development and perinatal period and activational effects on target organs in the adult state. Thus perinatal injection of testosterone or oestradiol into rats or mice affects the hypothalamus which regulates the gonadotrophin secretion of the pituitary in a feed back mechanism with gonads and pituitary (Harris, 1964). Injection of steroid hormones shortly after birth into female mice or rats suppresses the oestrus cycle (Barraclough, 1961; Kimura, Basu and Nandi, 1967; Kimura, Nandi and DeOme, 1967; Kimura and Nandi, 1967; Mori, 1967; Kimura and Takasugi, 1964; Takasugi, 1963; Takasugi and Kimura, 1964; Takasugi and Bern, 1964; Adams Smith and Peng, 1966; Takewaki, 1962; Takewaki and Mori, 1967) and leads to persistent oestrus or dioestrus depending on species, strain and dose of steroid administered. Persistent oestrus in rodents may occur spontaneously or may be induced by a variety of procedures and substances in the perinatal period: transplants of testis, continuous illumination or auditory stimulation, hypo thalamic lesions, parabiosis with gonadectomised rats, ligation of oviducts, injection of oestrogens, androgens, progesterone, deoxycorticosterone, cholesterol (Takewaki, 1962). The only common denominator as regards the injected substances in the perinatal period is the " gross disturbance in the concentration in the blood and perhaps androgenic metabolites produced as a by-product in some cases " (Harris, 1964).

Since cervico-vaginal tumours appear in old oestrogenised (Dunn and Green, 1963; Takasugi and Bern, 1964) as well as androgenised mice (Kimura and Nandi, 1967) and at least hyperplastic changes in rats (Takasugi and Kimura, 1964), it was considered worthwhile to test the influence on DMBA-induced carcinogenesis in the cervico-vaginal tract of administration of gonadal hormones in the perinatal period and to compare the effects with those of continuous and intermittent hormonal treatment of rats in the adult state (Glucksmann and Cherry, 1968). Continuous administration of testosterone and oestrogens affects carcinogenesis in different ways: in intact rats oestrogens inhibit the induction of sarcomas while testosterone merely prolongs the induction period. The induction of epithelial tumours is promoted by testosterone, but not by oestrogens given continuously. In castrate rats testosterone promotes and accelerates the appearance of sarcomas and of epithelial tumours, while oestrogens given continuously fail to promote carcinogenesis. The effect of oestrogens on carcinogenesis is quite independent of its stimulating action on stroma and epithelium of the cervico-vaginal tract. Intermittent administration in low doses fails to restore to normal the castrate

* Working with a grant from the British Empire Cancer Campaign for Research.
† Gibb Senior Fellow, British Empire Cancer Campaign for Research.

state of the normal tissues, but greatly promotes carcinogenesis. In view of these divergent actions of male and female gonadal hormones in the adult state, it is surprising to find that they have the same inductive actions in the perinatal period. There are, however, some indications that as regards onset of puberty at least, testosterone acts differently from oestrogen (Takewaki, 1962) and the same applies to the epithelial differentiation of the vagina of rats (Takewaki and Mori, 1967).

In the experiments to be reported here the differential action of androgens and oestrogens administered in the perinatal period was investigated as well as their influence on carcinogenesis induced by weekly applications of DMBA to the vaginal tract starting at about 2 months of age.

### MATERIALS AND METHODS

Hooded rats of the Lister strain random bred within a closed colony since 1940 were used for the experiments. The number of animals in the various treatment groups are given in Table I. Female and some male rats were injected subcutaneously within 24 hours of birth either with 0·07 mg. of oestradiol monobenzoate (Organon) or 1·25 mg. of testosterone propionate (Perandren, Ciba). In some groups the injections were repeated after 24 hours.

TABLE I.—*Treatment Groups, Survival Data, Incidence of Leukaemia and Pituitary Adenomas*

| | No. at risk | Survival in days: Range | Mean | Leukaemia % | Pituitary adenoma % |
|---|---|---|---|---|---|
| Oestradiol | | | | | |
| Group 1. 1x | 10 | 420–759 | 568 | 0 | 20 |
| „ 2. 2x + Acetone | 21 | 193–869 | 543 | 10 | 38 |
| „ 3. 2x + DMBA | 25 | 304–570 | 419 | 0 | 8 |
| „ 1–3 | 56 | 193–869 | 492 | 4 | 21 |
| Testosterone | | | | | |
| „ 4. 1x | 9 | 353–750 | 630 | 11 | 22 |
| „ 5. 2x | 27 | 111–776 | 546 | 15 | 50 |
| „ 6. 2x + DMBA | 21 | 193–459 | 332 | 5 | 14 |
| „ 4–6 | 57 | 111–776 | 485 | 14 | 33 |

For carcinogenic treatment a 1% solution in acetone of 9,10-dimethyl-1,2-benzanthracene (DMBA) was applied once weekly from the age of 6–8 weeks. The vagina was opened by dorsal flexion of the tail and the solution was distributed over the cervix, vagina and introitus by means of a cotton wool swab mounted on a thin wire rod. Control animals were painted similarly with acetone (Group 2).

Vaginal smears were collected with a moistened cotton wool swab and stained with haematoxylin-eosin. For fertility tests male and female litter mates injected once only with either testosterone or oestradiol were housed together throughout the duration of the experiment.

The rats were given food pellets and water *ad libitum* and housed 7 to a cage. Sick animals and those with clinical signs of vaginal and vulval tumours were killed, the vulva examined for clitoridal hypospadias and at post mortem the following tissues, in addition to those of the genital tract from ovary to vulva, were fixed: pituitary, thyroid, adrenals, lungs, liver, spleen, thymus, kidneys, intestine, mesenteric lymph nodes and salivary glands. The material was fixed in Zenker-acetic or Bouin's fluid, dehydrated, embedded in paraffin, sectioned at 6–8 $\mu$ and stained with haematoxylin-eosin, Van Gieson, carmalum-orange G-aniline blue, Southgate's mucicarmine or the periodic acid-Schiff technique (PAS) after diastase digestion.

### RESULTS

*Effects of testosterone and of oestrogens on fertility and on the external genitalia:*

(1) *Opening of the vaginal orifice.*—Single administration of oestradiol to newly born rats advanced the time of vaginal opening; the first animal with an open introitus was found on the 10th day, the last on the 22nd day and the median time for opening of the vagina was 14 days. Two injections of oestradiol had the same effect of accelerating puberty.

With single applications of testosterone a third of the rats had closed vaginas even at 40 days, which remained closed throughout life. With 2 injections of testosterone 16 of 21 vaginas were still closed on the 66th day when they had to be opened for the application of DMBA. Of the control rats injected twice perinatally with testosterone 60% had closed vaginas throughout life.

(2) *Changes in vaginal smears.*—At 4 months repeated smears were taken from all animals with an open vagina following single injections of oestradiol or testosterone. Of 10 females given oestradiol 2 only had persistent oestrus, while the others had signs of irregular cyclical changes. One of the persistent oestrus females had a litter before and one after that time. Of the 6 testosterone treated animals with an open vagina only 1 had persistent oestrus and was sterile.

(3) *Fertility of rats given single injections perinatally.*—The injected females were kept with their injected male litter mates and the times at which litters were produced and the number of offspring are recorded in Fig. 1. After testosterone treatment 4 rats had several and one a single litter. Of the 4 sterile rats 3 had closed vaginas throughout life. The 5 fertile rats had 142 babies in 17 litters, i.e. an average of 8·3 per litter. Of the 5 fertile rats treated with oestradiol 1 had 2 litters, while the others had one only. The total number of babies was 34 in 6 litters, i.e. an average of 5·7.

Of 6 oestrogenised males killed at age 630–759 days 4 showed active spermatogenesis and the other 2 had impaired spermatogenesis. Two of 3 males of the same age, but treated perinatally with testosterone, also had somewhat impaired spermatogenesis.

While the difference in number and size of litters between perinatally oestrogen- and testosterone-treated animals might have been due to an effect of oestrogens on fertility of males, there was no histological evidence for an action on the spermatogenesis additional to ageing.

(4) *Hypospadias and formation of the os clitoridis.*—Hypospadias in the form of cleft clitoris with the urethra opening in the vagina instead of at the tip of the clitoris was seen in rats treated perinatally with testosterone or with oestradiol, though with different frequency. In testosterone treated females hypospadias occurred also when the vagina appeared closed. The incidence in control animals given two perinatal injections is indicated in Table II and shows a significant difference in favour of oestrogen administration.

The clitoris was greatly enlarged in rats injected perinatally with testosterone. The corpora cavernosa were distended and so was the central tissue with enlarged

FIG. 1.—Litters produced by rats given single perinatal injections of osteradiol or testosterone. The number of offspring is given in the boxes for each litter timed from the day of birth of the mother.

sinuses. In the median area the connective tissue formed a fibrocartilaginous mass which later ossified (Fig. 2 and 3). The incidence of the bony formations of the clitoris is given in Table II and may err on the low side as they were diagnosed in histological preparations and not all vulvas were sectioned in the correct plane. None were seen in rats treated perinatally with oestradiol, nor was the clitoris enlarged in these animals.

*Effects of testosterone and of oestrogens on survival of rats, on the incidence of leukaemia and of pituitary adenomas, on the breast, salivary glands and liver:*

For the purpose of this paper only animals surviving for at least 100 days were considered " at risk ", though a few controls were killed before that time and not included in Table I. Except for rats additionally treated with DMBA, the survival periods of oestrogenised and androgenised rats were fairly similar. The exceptions

TABLE II.—*Effects of Testosterone and Oestradiol given Perinatally to Female Rats*

| Organs | Groups | Testosterone (%) | Oestradiol (%) |
|---|---|---|---|
| *Breast:* hyperplasia | 1–6 | 54 | 15 |
| tumours | 1–6 | 7 | 0 |
| *Ovary:* abortive luteinisation | 1–6 | 46 | 2 |
| abscess (no DMBA) | 1, 2, 4, 5 | 13 | 26 |
| (with DMBA) | 3, 6 | 52 | 24 |
| *Uterus:* enlarged | 1–6 | 53 | 22 |
| atrophic | 1–6 | 28 | 39 |
| normal | 1–6 | 19 | 39 |
| squamous metaplasia | 1–6 | 45 | 28 |
| *Vulva:* hypospadias | 2, 3, 5, 6 | 22 | 71 |
| os clitoridis | 1–6 | 25 | 0 |
| *Salivary glands:* male type | 1–6 | 63 | 39 |
| female type | 1–6 | 4 | 14 |
| intermediate | 1–6 | 33 | 47 |
| *Liver:* hepatomas and cholangiomas | 1–6 | 9 | 2 |

were due to a high incidence of abscesses in the ovary and oviduct (cf. below and Table II), which made it necessary to kill the animals.

The leukaemias were of the same type as those described for control intact and spayed rats of our colony born at the same time as the animals of the present series. In the intact and castrate controls the incidence was 25% in 20 animals of each group; in perinatally injected rats the leukaemia incidence (Table I) was thus reduced particularly with oestrogen administration. It is noteworthy that additional treatment with DMBA did not increase the incidence.

Pituitary adenomas occurred frequently in aged rats. In our control series (Glucksmann and Cherry, 1968) we found 7 in 20 intact and 11 in 20 castrate females. In the latter hyperplasia and hypertrophy of gonadotrophs and castration cells were observed regularly. In the perinatally treated animals gonadotrophs were present and occasionally were numerous and hypertrophic but castration cells were not seen.

In our control series the incidence of breast tumours in intact rats varied from 7% in 1955, 1956 to 10% in 1964, while none occurred in spayed females. The incidence of 7% breast tumours in the animals injected perinatally with testosterone thus falls within the control range while that of oestrogenised rats is similar to that of castrates. Hyperplasia of the breast in the form of duct and acinar proliferation associated with secretory activity was considerably increased in androgenised rats (Table II), and often led to the appearance of cysts.

Rats of our colony have a marked sex dimorphism in the submaxillary gland indicated by the greater volume and secretory activity of the tubules in males. This dimorphism is revealed also in the response of the glands to tumour induction by the local administration of DMBA (Glucksmann and Cherry, 1966). In both androgenised and oestrogenised females the volume and secretory activity of the tubules were increased particularly by perinatal administration of testosterone (Table II).

Especially in androgenised old females the incidence of hepatomas and cholangiomas was increased over the control level as well as over that of oestrogenised animals (Table II).

*Effects of testosterone and of oestrogen on ovaries, uterus and cervico-vaginal tract:*

(1) *Ovaries.*—In old untreated control animals the ovaries often lack corpora lutea and in our series this was found in 65% of rats aged 400–765 days. None of the animals injected twice perinatally with testosterone or oestrogen had corpora lutea. After a single injection corpora lutea were seen in one animal of each group at 353 and 654 days. Since 5 animals of each group produced litters (cf. above), it must be assumed that they had corpora lutea at the time of pregnancy (Fig. 1).

Abscesses involving the ovaries, the oviduct and at times the uterine horns occurred in 18 of 56 (32%) androgenised and in 16 of 56 (29%) oestrogenised rats. The incidence varied from 60% of rats given a single injection of oestradiol and 52% of animals given 2 doses of testosterone and painted additionally with DMBA to 11% for a single dose of testosterone and 19% for two doses of oestradiol given perinatally. There is thus no clear evidence of a differential action of testosterone and oestradiol in causing these abscesses.

In androgenised as well as in oestrogenised rats the ovaries were fairly small and contained ova, primordial, some Graafian and very many atretic follicles. Frequently these were deformed and gave rise to masses of atretic cells, often described as " hyperplasia of interstitial tissue ". In animals treated perinatally with either of the steroids the characteristic cell of the atretic follicles and masses was fairly large with vacuolar " empty " cytoplasm and a nucleus with coarse chromatin clumps (Fig. 4). In androgenised rats in some of the follicles the central cells revealed an abortive attempt at luteinisation (Fig. 5 and Table II) as evidenced by their enlargement, the appearance of denser more granular cytoplasm and of larger nuclei with evenly distributed chromatin. Larger cells with intensely PAS-positive granulation were seen in the centre of atretic follicles independently of abortive luteinisation.

In addition to the atretic follicles and masses the ovaries of old animals contained a number of tubules and follicles derived from invaginating germinative epithelium which was of a columnar type particularly in testosterone-treated rats and which also evaginated to form papilliform excrescences and adenomata. The cells of the invaginated follicles enlarged, adopted a high columnar shape and almost filled the central space which in normal primordial tubules and follicles would have been occupied by an ovum. These formations might be considered as abortive primary follicles.

(2) *Uterine horns.*—In normal animals the diameter of the cross section of uterine horns varied with oestrus cycle around a diameter of 1·5 mm. in the histological specimen. Roughly the same size was found in about 20% of the rats treated perinatally with testosterone and in 40% of the oestradiol-treated group. A marked enlargement was more frequent in androgenised than in oestrogenised rats (Table II) while a reduction in size occurred more frequently after oestradiol than after testosterone treatment. The reduction, however, was never similar to that induced by castration. Changes in size affected the endo- as well as the myometrium, and the endometrial glands. All the various groups (single or double injections of the hormones, with or without additional painting with DMBA) showed the differences reflected in the figures given in Table II with only minor variations in the percentages.

Areas of squamous metaplasia of the endometrial epithelium (Fig. 6) or some of the glands (Fig. 7) appeared more frequently in testosterone- than in the oestrogen-treated rats (Table II). Such squamous changes in the glands often co-existed with the presence of a high columnar endometrial epithelium (Fig. 7). In some of the androgenised rats a high secretory epithelium was associated with adenomatous glandular hyperplasia (Fig. 8) and in others with the appearance of a stratified columnar secretory epithelium at the mouth of the glands.

Uterine abscesses and pus in the uterine lumen were seen in 25% of the androgenised and in 21% of the oestrogenised rats. These inflammatory lesions were sometimes, but by no means always associated with squamous metaplasia of the uterus. The uterine and ovarian abscesses were not closely related. Of 51 rats with abscesses in either uterus or ovaries only 14 (27%) had lesions in both organs, 24 (47%) in ovaries only and 13 (26%) in the uterine horns only and the same figures were obtained for animals treated with either testosterone or oestradiol in the perinatal period.

(3) *Cervico-vaginal tract.*—Since DMBA was administered to this region the description of hormonal effects is restricted to single and double perinatal applications of testosterone and oestradiol, but includes the series of oestrogenised rats painted once weekly with acetone. The reaction varied from the cervical to the vulval end of the vagina. At the posterior end stratified epithelium usually with cornification was nearly always found. The cervical lining varied from a high columnar, mucin secreting structure to a low cuboidal secretory or to a stratified often keratinising epithelium. The same held for the intermediate region though its structure might have differed in any given rat from that of the anterior region. The vaginal smear is thus only a rough indication of the status of the vaginal epithelium in its entirety (Adams Smith and Peng, 1966; Takewaki and Mori, 1967).

---

### EXPLANATION OF PLATES.

FIG. 2 and 3.—Os clitoridis in a female aged 391 days injected twice with testosterone within 48 hours of birth. Note the replacement of cartilage by bone. Van Gieson ×100; ×195.

FIG. 4.—Atretic follicle in ovary of a 784 days old rat injected twice within 48 hours of birth with oestradiol and 2 months later given weekly paintings with acetone of the cervico-vaginal tract. PAS. ×425.

FIG. 5.—Atretic follicle in ovary of a 737 days old rat injected twice within 48 hours of birth with testosterone. The enlarged central cells suggest abortive attempts at luteinisation. PAS. ×360.

FIG. 6.—Squamous metaplasia in the lining of the uterine horn of a 270 days old androgenised rat, given weekly DMBA paintings of the cervico-vaginal tract from 2 months on. H. & E. ×77.

FIG. 7.—Squamous metaplasia in an endometrial gland and high columnar epithelium of the endometrium in a 459 days old androgenised rat given weekly DMBA-paintings of the cervico-vaginal tract from 2 months on. PAS. ×160.

FIG. 8.—Adenomatous hyperplasia of endometrial glands in a 452 days old androgenised rat given weekly DMBA-paintings of the cervico-vaginal tract from 2 months on. PAS. ×132.

FIG. 9.—Squamous cell carcinoma extending almost throughout the width of the upper vagina in a 691 days old oestrogenised rat given weekly acetone-paintings of the cervico-vaginal tract from 2 months on. PAS. ×100.

Fig. 10.—Same animal as in Fig. 9. The tumour has spread along the perineural lymphatics and forms a sheath (a) around a ganglion at the cervico-vaginal border. PAS. ×125.

FIG. 11.—Mixed carcinoma in the upper vagina of a 425 days old oestrogenised rat treated with acetone like that of Fig. 9. Mucin secreting foci (m) as well as squamous formations are seen. PAS. ×200.

FIG. 12.—Same animal as in Fig. 11. Tumour cells (a) have invaded the adjacent muscle. PAS. ×200.

2

3

4

5

Cherry and Glucksmann.

Cherry and Glucksmann.

Excluding the posterior section, testosterone treatment resulted in a cornifying epithelium throughout the vagina in 57% of rats, in secretory epithelium throughout in 27% and in columnar secretory epithelium near the cervix and cornifying epithelium in the intermediate region in 15%. The corresponding figures for oestrogen treatment were 71%, 10% and 19%. There was thus a slightly greater tendency to promote cornification with oestrogen than with testosterone administration.

The most striking proliferative changes in the cervico-vaginal epithelium were induced in rats twice given oestradiol in the perinatal period and subsequently once weekly paintings with acetone to the genital tract. Hyperplastic changes resulted at first in radication, i.e. projections of the epithelium into the supporting connective tissue, of varying extent and area and later in the appearance of extruding as well as intruding papillomata and even of carcinomata. Similar premalignant and malignant changes in oestrogenised mice were reported without the use of acetone (Dunn and Green, 1963; Kimura and Nandi, 1967; Takasugi and Bern, 1964), while the use of acetone in intact and castrate rats and mice failed to induce premalignant or malignant proliferation (Glucksmann and Cherry, 1968; Murphy, 1961; v. Haam and Scarpelli, 1955).

The incidence of epithelial tumours is given in Fig. 15 and 16 (oestradiol + acetone). Some of the tumours were squamous cell carcinomas (Fig. 9) which extended into the deeper layers of the stroma and muscle (Fig. 12) and grew along the perineural lymphatics to the ganglia (Fig. 10). Others were of the mixed type (Fig. 11) containing a squamous as well as a secretory columnar component. In the oestrogenised controls no tumours of stromal origin occurred in the cervico-vaginal tract and no epithelial tumours in the vulva.

In androgenised rats without DMBA treatment no epithelial tumours were found in either the cervico-vaginal tract or the vulva. One leiomyofibrosarcoma and one leiomyofibroma of the upper vagina were observed after 580 and 776 days respectively.

*Effects of weekly DMBA paintings on the induction of tumours in the cervico-vaginal tract:*

Since the painting of oestrogenised and androgenised rats was started at the same time (2 months of age) as in intact and castrate animals and the first application of DMBA was taken as the start of all experiments, the findings on tumour induction are strictly comparable. Sarcomas were of various types (Glucksmann and Cherry, 1968) and similar in all the groups, though they differed in rate of development and incidence. The cumulative percentage incidence is plotted for the 4 groups in Fig. 13 which indicates that perinatal treatment with testosterone or oestradiol causes an even greater delay in tumour induction than that due to castration, and a total incidence like that in spayed rats. The rate of tumour induction appeared to be similar in androgenised and oestrogenised animals.

A difference in incidence of sarcomas between androgenised and oestrogenised rats became apparent when the age-specific induction rates were calculated for periods of multiples of 100 days. The percentage of the animals at risk is plotted at the mid-point of the 100 day period in Fig. 14. Both types of perinatal treatments delayed the onset of tumour induction by at least 100 days. Subsequently, however, in rats treated with testosterone the rate of tumour induction resembled that of intact animals while in those given oestradiol perinatally it was similar to that of the castrates. (The numbers in the graph indicate the number of rats surviving for the later periods of the experiment. For reasons of clarity the initial induction rate for sarcomas in castrate rats is drawn to 100 instead of 150 days; the correct induction time is given in Fig. 13.)

FIG. 13.—Cumulative percentage incidence of cervico-vaginal sarcomas induced by DMBA in intact, castrate, androgenised and oestrogenised rats.

The cumulative incidence of epithelial tumours is plotted in Fig. 15. Carcinomas and papillomas were not separated as the numbers were very small. The proportions of carcinomas to all epithelial tumours was as follows:—

| | |
|---|---|
| Intacts | 1/3 = 33% |
| Castrates | 0/4 = 0% |
| Androgenised | 1/4 = 25% |
| Oestrogenised | 5/12 = 42% |
| Oestrogenised + acetone | 4/9 = 44% |

The time of appearance of carcinomas and of papillomas overlapped throughout the experimental period. As mentioned above, the control oestrogenised rats painted with acetone had an appreciable incidence of epithelial tumours and amongst them the proportion of carcinomas to papillomas was similar to that in oestrogenised animals treated with DMBA. While only a few epithelial tumours occurred in intact, castrate and testosterone-treated rats, oestradiol promoted the induction of epithelial tumours. This is seen more clearly in Fig. 16 in which the age-specific rates are plotted. Oestradiol perinatally followed by DMBA applied weekly had a continuously rising induction rate with age and numbers of applications of DMBA

which was clearly distinct from the testosterone group and also from the acetone-treated oestrogenised rats. Thus looked at from the age-specific rates the perinatal testosterone treatment induced more sarcomas but fewer epithelial tumours than the perinatal oestrogenisation. Both perinatal treatments delayed the onset of epithelial and connective tissue tumours as compared with intact and castrate animals.

FIG. 14.—Age-specific induction rates of cervico-vaginal sarcomas induced by DMBA in intact, castrate, androgenised and oestrogenised rats.

### DISCUSSION

Perinatal administration to female rats of either testosterone or oestradiol suppresses or renders highly irregular the oestrous cycle, inhibits the formation of corpora lutea, reduces or abolishes the fertility depending on dosage, induces uterine and ovarian abscesses and delays the induction by DMBA of tumours in the cervico-vaginal tract. Androgenisation delays the opening of the vagina (Selye, 1940; Bradbury, 1941; Takasugi, 1963; Takewaki, 1962; present experiments) while puberty is precocious in oestrogenised rats (Takasugi and Kimura, 1964; present experiments). At different dose levels and for prolonged periods of testosterone treatment puberty may be precocious also in androgenised rats (Segal and

Johnson, 1959; Harris, 1964), suggesting that the basic inductive actions of oestrogens and androgens on the developing hypothalamus may be similar depending on dosage of the hormone. The same may apply to the induction of hypospadias of the clitoris which with our treatment occur more frequently in oestrogenised than in androgenised rats, while in mice they appear with equal frequency (Kimura, Basu and Nandi, 1967). On the other hand an increase in growth rate of females has been reported only after perinatal testosterone treatment (Harris, 1964). Similarly we have never seen the formation of an os clitoridis in oestrogenised rats, while it occurs in females, treated perinatally or even after puberty with testosterone

FIG. 15.—Cumulative percentage incidence of cervico-vaginal papillomas and carcinomas induced by DMBA in intact, castrate, androgenised and oestrogenised rats and in oestrogenised controls painted with acetone.

(present experiments; Glucksmann and Cherry, 1968). In other respects there are significant quantitative differences between oestrogenised and androgenised rats: abortive luteinisation, hypertrophy of the uterus and squamous metaplasia, enlargement of secretory tubules and activity in the submaxillary gland predominate in androgenised rats, while epithelial hyperplasia of the cervix and vagina and cornification do so in oestrogenised animals (cf. also Takewaki and Mori, 1967, for mice; Takasugi and Kimura, 1964, for rats). In our experiments tumour formation in the cervico-vaginal epithelium without additional carcinogenic stimulation is found in oestrogenised, but not androgenised rats. In mice Kimura and Nandi (1967) report the induction of hyperplastic and possibly neoplastic lesions of the cervix and vagina at 15–17 months in animals given oestradiol or testosterone with about the same frequency, and a significantly lower incidence in animals treated with gonadal hormones perinatally, but ovariectomised at 100–120 days.

The induction by weekly applications of DMBA of tumours in the cervico-vaginal tract is delayed by perinatal administration of testosterone as well as of oestrogens and this prolongation of the induction period is greater even than in spayed rats (Fig. 13 and 15). The cumulative percentage incidence of vaginal sarcomas is of the same order for castrate, androgenised and oestrogenised rats (Fig. 13), while for epithelial tumours it is raised above the level of intact and castrate rats in androgenised and considerably more so in oestrogenised animals (Fig. 15). Even the oestrogenised controls painted with acetone have considerably more tumours than intact, castrate or androgenised females treated with DMBA.

Fig. 16.—Age-specific induction rates of cervico-vaginal papillomas and carcinomas induced by DMBA in androgenised and oestrogenised rats and in oestrogenised controls painted with acetone.

If the age-specific induction rates are considered, the picture is changed: intact and androgenised rats show a marked increase of sarcomas with age and thus with dosage of carcinogen (Fig. 14), while there is no such age- and dose-related increase for castrate and oestrogenised females. For epithelial tumours there is no age- and dose-related increase in rate of carcinogenesis for intact and spayed rats and at a higher level for androgenised DMBA-treated and oestrogenised control animals (Fig. 16). Oestrogenised females, on the other hand, show a great increase in tumour induction related to dose and age. There is thus a striking difference in the responsiveness of the connective tissue and the epithelium of the cervicovaginal tract in androgenised and oestrogenised animals, though in both the induction time and dosage of carcinogen is increased over the level necessary for intact and castrate animals. Furthermore in the same animals the induction of predominantly epithelial tumours in the vulva is the same for intact, castrate, androgenised and oestrogenised rats whether measured by duration of induction period, cumulative percentage incidence or age- and dose-related induction rates. These findings are in contrast with the high frequency of hypospadias in oestrogenised and the induction of bone in androgenised animals, neither of which phenomena is seen in intact or castrate rats. For the vulva as well as the cervico-vaginal tract the hormonal action on carcinogenesis is thus quite distinct from that on the normal tissues, whether given perinatally or to the adult animal.

Since persistent oestrous conditions of the cervico-vaginal canal have been taken to imply raised levels of continuous oestrogen secretion (Barraclough, 1961), it is interesting to compare the effect of perinatal oestrogen and testosterone treatment on carcinogenesis with that of the administration of the gonadal hormones to adult rats. Table III summarises the results previously reported (Glucksmann and Cherry, 1968) and those of the present experiments. The comparison is made with intact and castrate rats respectively treated solely with weekly applications of DMBA to the cervico-vaginal tract. Intermittent oestrogenic treatment implies adding stilboestrol to the drinking water on 3 consecutive days per week, while continuous treatment means daily administration of stilboestrol in the drinking water which in respect of carcinogenesis and other actions has been equal to 2 intramuscular injections of 1·5 $\mu$g. of oestradiol monobenzoate in oil. Testosterone propionate has been administered by depots in the subcutis. The induction time for sarcomas and epithelial tumours in oestrogenised and androgenised rats is increased more than in intact rats treated continuously with either oestrogens or testosterone and this implies that the number of weekly applications of DMBA also is greater. The incidence of sarcomas is reduced to the same level by castration, by oestrogen treatment of the adult and the newborn rat. The cumulative percentage incidence of sarcomas of androgenised rats is also equal to that of castrate animals (indicated by the minus sign in brackets in Table III), but the

TABLE III.

| | Sarcomas | | Epithelial tumours | |
|---|---|---|---|---|
| Treatment | Time | Incidence | Time | Incidence |
| Oestrogens: | | | | |
| perinatal ♀ | − | − | − | + |
| intermittent ♀ | = | = | = | = |
| continuous ♀ | − | − | = | = |
| intermittent ♀̸ | + | + | + | + |
| continuous ♀̸ | = | = | = | = |
| Testosterone: | | | | |
| perinatal ♀ | − | =(−) | − | = |
| continuous ♀ | − | = | = | + |
| continuous ♀̸ | + | + | = | + |

−, indicates longer induction time and lower incidence of tumours.
+, indicates shorter induction time and greater incidence of tumours.
=, indicates no changes in induction time or incidence of tumours.

age-related induction rate equals that of intact rats. None of the hormones administered to the adult females has the same effect on carcinogenesis as has perinatal treatment. As regards sarcomas the cumulative percentage incidence in castrates is similar to that in females with persistent oestrus. For epithelial tumours the incidence in androgenised rats is similar to that in testosterone-treated intact and castrate adults but significantly less than in the oestrogenised animals; these in turn have fewer papillomas and carcinomas than castrates given stilboestrol intermittently (i.e. 74% over 271 days) in which the uterus remains of castrate dimensions.

The carcinogenesis in the female genital tract cannot be correlated with the number and activity of gonadotrophs in the pituitary. In old rats pituitary adenomas appear quite frequently and are seen also in oestrogenised and androgenised rats, but bear no correlation with the induction of either sarcomas or epithelial tumours. In castrates the gonadotrophs are hypertrophic, increased in number and give rise to castration cells. Castration cells are not found in our perinatally treated rats, but there is some hypertrophy and hyperplasia of gonadotrophs. This is contrary to the report of Kawashima and Takewaki (1966), quoted by Mori (1967), of a decrease of gonadotrophs in persistent oestrus and absence in persistent dioestrous rats. The length of the observation periods in the different experiments may account for this discrepancy. The difference between androgenised and oestrogenised animals in carcinogenesis as well as gain in weight, appearance of the os clitoridis, prevalence of hypospadias and abortive luteinisation makes it unlikely that all the deviations from the behaviour of the intact animals can be accounted for by differences in the rhythm and level of gonadotrophin secretion due to identical hypothalamic lesions. These may differ themselves in animals perinatally treated with oestrogens and testosterones. It is interesting that reserpine acting on the hypothalamus of adults may alter the rate of tumour induction in rats and some strains of mice though not necessarily in the same direction (Lacassagne, 1961).

Old rats are prone to the appearance of endocrine adenomas particularly of the pituitary, thyroid, adrenal and ovary. In these old animals tumours of the cervico-vaginal tract are very rare, possibly because of the slow development of the adenomas which restricts their carcinogenic action on the vagina and cervix to a short fraction of the survival period. Even in oestrogenised animals the appearance of epithelial tumours in controls does not increase with age (Fig. 16) in contrast to oestrogenised animals treated with DMBA. This observation underlines the fact that hormonal factors are by themselves weakly carcinogenic, but potent modifiers of the carcinogenic process induced by chemical carcinogens.

### SUMMARY

1. Perinatal treatment of female rats with either testosterone or oestradiol prolongs the induction period for DMBA-induced tumours in the cervico-vaginal tract as compared with intact and castrate animals.

2. The total incidence of vaginal sarcomas is similar for both types of perinatal treatment and resembles that in spayed rats. Oestradiol treatment promotes the induction of epithelial tumours even in control animals painted with acetone; testosterone, though less effective, also raises the percentage incidence above that of intact and castrate females.

3. Androgenised like intact, but not castrate or oestrogenised rats show an age- and dose-related increase in rate of sarcoma induction. The rate of induction of epithelial tumours is related to age and dose in oestrogenised females only.

4. Abortive luteinisation, squamous metaplasia in the uterus and male characteristics in the secretory tubules of the submaxillary gland predominate in androgenised females while epithelial hyperplasia and cornification of the cervix and vagina and hypospadias of the clitoris do so in oestrogenised animals. Enlargement of the clitoris and the appearance of the os clitoridis occur only in testosterone treated animals.

5. These results are discussed in comparison with the effects of oestrogen and testosterone administration to adult females on the induction of cervico-vaginal tumours.

### REFERENCES

Adams Smith, W. N. and Peng, M. T.—(1966) *J. Physiol.*, **185**, 655.
Barraclough, C. A.—(1961) *Endocrinology*, **68**, 62.
Bradbury, J. T.—(1941) *Endocrinology*, **28**, 101.
Dunn, T. and Green, A. W.—(1963) *J. natn. Cancer Inst.*, **31**, 425.
Glucksmann, A. and Cherry, C. P.—(1966) *Br. J. Cancer*, **20**, 760.—(1968) *Br. J. Cancer*, **22**, 545.
v. Haam, E. and Scarpelli, D. G.—(1955) *Cancer Res.*, **15**, 449.
Harris, G. W.—(1964) *Endocrinology*, **75**, 627.
Kawashima, S. and Takewaki, K.—(1966) *Annotnes zool. jap.*, **39**, 23.
Kimura, T., Basu, S. L. and Nandi, S.—(1967) *J. exp. Zool.*, **165**, 71.
Kimura, T. and Nandi, S.—(1967) *J. natn. Cancer Inst.*, **39**, 75.
Kimura, T., Nandi, S. and DeOme, K. B.—(1967) *J. exp. Zool.*, **165**, 211.
Kimura, T. and Takasugi, N.—(1964) *J. Fac. Sci. Tokyo Univ.*, IV, **10**, 391.
Lacassagne, A.—(1961) *Presse méd.*, **51**, 2285.
Mori, T.—(1967) *J. Fac. Sci. Tokyo Univ.*, IV, **11**, 243.
Murphy, E. D.—(1961) *J. natn. Cancer Inst.*, **27**, 611.
Segal, S. J. and Johnson, D. C.—(1959) *Archs Anat. microsc. Morph. exp.*, **48**, 261.
Selye, H.—(1940) *Endocrinology*, **27**, 657.
Takasugi, N.—(1963) *Endocrinology*, **72**, 607.
Takasugi, N. and Bern, H. A.—(1964) *J. natn. Cancer Inst.*, **33**, 855.
Takasugi, N. and Kimura, T.—(1964) *J. Fac. Sci. Tokyo Univ.*, IV, **10**, 381.
Takewaki, K.—(1962) *Experientia*, **18**, 1.
Takewaki, K. and Mori, T.—(1967) *J. Fac. Sci. Tokyo Univ.*, IV, **11**, 193.